# Cortical gamma-band resonance preferentially transmits coherent input

**DOI:** 10.1016/j.celrep.2021.109083

**Published:** 2021-05-04

**Authors:** Christopher Murphy Lewis, Jianguang Ni, Thomas Wunderle, Patrick Jendritza, Andreea Lazar, Ilka Diester, Pascal Fries

**Affiliations:** 1 Ernst Strüngmann Institute (ESI) for Neuroscience in Cooperation with Max Planck Society, Deutschordenstraße 46, 60528 Frankfurt, Germany; 2 Brain Research Institute, University of Zurich, Winterthurerstrasse 190, 8057 Zurich, Switzerland; 3 International Max Planck Research School for Neural Circuits, Max-von-Laue-Straße 4, 60438 Frankfurt, Germany; 4 Donders Institute for Brain, Cognition and Behaviour, Radboud University Nijmegen, Kapittelweg 29, 6525 EN Nijmegen, the Netherlands; 5 Present address: Department of Neuroscience, Tufts University School of Medicine, 136 Harrison Ave., Boston, MA 02111, USA; 6 Present address: Optophysiology, Bernstein Center and BrainLinks-BrainTools, University of Freiburg, Albertstraße 23, 79104 Freiburg, Germany; 7 These authors contributed equally; 8 Lead contact

## Abstract

Synchronization has been implicated in neuronal communication, but causal evidence remains indirect. We use optogenetics to generate depolarizing currents in pyramidal neurons of the cat visual cortex, emulating excitatory synaptic inputs under precise temporal control, while measuring spike output. The cortex transforms constant excitation into strong gamma-band synchronization, revealing the well-known cortical resonance. Increasing excitation with ramps increases the strength and frequency of synchronization. Slow, symmetric excitation profiles reveal hysteresis of power and frequency. White-noise input sequences enable causal analysis of network transmission, establishing that the cortical gamma-band resonance preferentially transmits coherent input components. Models composed of recurrently coupled excitatory and inhibitory units uncover a crucial role of feedback inhibition and suggest that hysteresis can arise through spike-frequency adaptation. The presented approach provides a powerful means to investigate the resonance properties of local circuits and probe how these properties transform input and shape transmission.

## In brief

Rhythmic synchronization has been implicated in neuronal communication, yet causal evidence has remained scarce. Lewis et al. optogenetically stimulate the visual cortex to emulate synaptic input while recording spike output. Cortex resonates at the gamma band (30–90 Hz) and preferentially transmits input that is coherent to the ongoing gamma-band rhythm.

## Graphical abstract

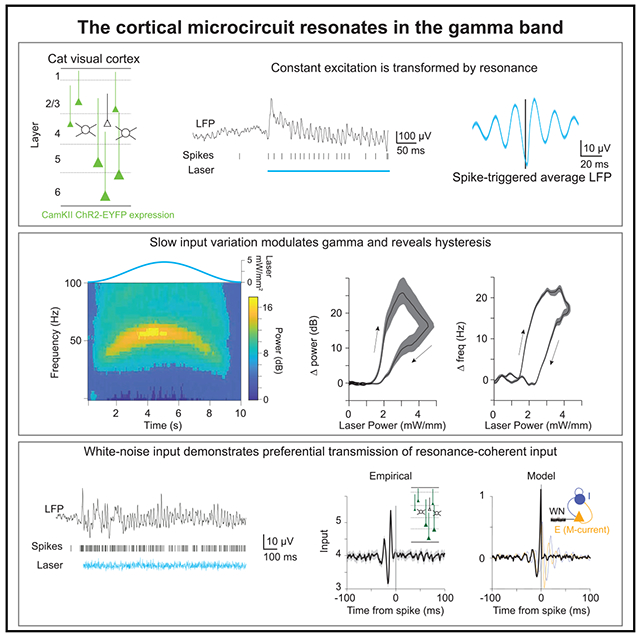

## INTRODUCTION

The brain’s computational abilities arise from communication within and between neuronal groups, and the dynamic modulation of neuronal communication is believed to enable flexible behavior (Engel et al., 2001; Fries, 2015; Varela et al., 2001). A compelling means to modulate neuronal communication is synchronization (Akam and Kullmann, 2010; Azouz and Gray, 2003; Börgers and Kopell, 2008; Hahn et al., 2014; Palmigiano et al., 2017; Salinas and Sejnowski, 2001; Wang, 2010). Neuronal synchronization is determined by cellular and network properties that define intrinsic timescales for activity. The intrinsic timescale of cells and circuits can be characterized by resonance, i.e., how inputs are amplified, or preferentially transmitted. In single neurons, specific combinations of diverse conductances can establish membrane and firing-rate resonances (Fellous et al., 2001; Hutcheon and Yarom, 2000; Lampl and Yarom, 1997; Schreiber et al.,2004). In networks, interactions between recurrently coupled excitatory and inhibitory (E-I) neurons generate resonances based on connectivity (Börgers and Kopell, 2003; Buzsáki and Wang, 2012; Tiesinga and Sejnowski, 2009; Whittington and Traub, 2003).

A prominent cortical resonance occurs in the gamma band (30–90 Hz) (Adesnik and Scanziani, 2010; Cardin et al., 2009; Etter et al., 2019; Iaccarino et al., 2016; Lu et al., 2015; Ni et al., 2016; Sohal et al., 2009). The Communication-through-Coherence (CTC) hypothesis (Fries, 2005, 2015) proposes that gamma-band synchronization between neuronal groups can flexibly determine their communication. Computational models have demonstrated that gamma-rhythmic inputs can entrain a postsynaptic population of recurrently coupled E-I units, thereby enhancing the impact of the entraining input and reducing the impact of competing inputs (Börgers and Kopell, 2008; Hahn et al., 2014; Palmigiano et al., 2017). This proposal has accrued considerable correlative evidence, for example, gamma-rhythmic gain modulation of neuronal and behavioral responses (Ni et al., 2016); phase-dependent power-covariation and transfer entropy between neuronal groups (Besserve et al., 2015; Womelsdorf et al., 2007); and selective enhancement of interareal gamma-band synchronization by attention (Bosman et al., 2012; Grothe et al., 2012), which improves behavioral performance (Rohenkohl et al., 2018). However, it has remained difficult to provide direct causal evidence for selective transmission of coherent inputs via network resonance.

Direct evidence for a causal role of synchronization in neuronal communication can be obtained through experimental control of network input and simultaneous measurement of spike output (Akam et al., 2012). We emulated excitatory synaptic input to a local population with millisecond temporal precision using Channelrhodopsin-2 (ChR2), a light-activated cation channel (Boyden et al., 2005). We transfected pyramidal cells in the cat visual cortex, a classical model for investigating cortical information processing (Douglas and Martin, 2004). Illumination of ChR2-expressing neurons enabled control of excitation *in vivo*.

Stimulation with constant light confirmed the previous finding that cortical networks can transform temporally flat excitatory input into gamma-rhythmic spike output (Adesnik and Scanziani, 2010; Lu et al., 2015; Ni et al., 2016) with features similar to that generated by visual stimulation (Fries et al., 1997, 2002; Gray et al., 1989; Gray and Viana Di Prisco, 1997). Slowly varying the excitation to the network with ramps and symmetric stimulation profiles revealed that the peak frequency of the gamma resonance could vary between 30 and 70 Hz, and that there was pronounced hysteresis for both the power and the frequency. Sinusoidal stimulation demonstrated that network spike output was entrained by rhythmic input with a fidelity that increased up to 40 Hz and decreased slightly for 80 Hz.

Finally, we sought to determine if the intrinsic resonance of visual cortical populations can act as a filter to select coherent components of external excitatory drive. Direct stimulation of excitatory cells with temporal white noise dramatically illustrated that the resonant properties of the local circuit established an endogenous temporal receptive field, or window of opportunity, for external excitatory drive. In contrast with periodic signals (like sinusoids or rhythmic pulse-trains), white noise is not auto-correlated and, therefore, enables a causal analysis of network transmission, i.e., from excitatory input to spike output (Bryant and Segundo, 1976; Mainen and Sejnowski, 1995; Marmarelis and Naka, 1972). Spike-triggered averaging of the white-noise light sequence revealed that spikes were preceded by episodes of gamma-rhythmic input. Correspondingly, an analysis of Granger causality between the white-noise input and neuronal spike output revealed a pronounced gamma-band peak. Simulations with a well-established recurrent network composed of conductance-based model neurons (Börgers, 2017) reproduced our core results. Modeling confirmed the central role of strong, fast feedback inhibition in gamma-band resonance (Börgers and Kopell, 2003; Sohal et al., 2009; Stark et al., 2014). The essential resonance phenomena were also evident in a greatly simplified network of leaky-integrate-and-fire (LIF) units. Modeling of the power and frequency hysteresis effects required the addition of a non-inactivating potassium current, the M-current, to the excitatory units. Overall, our results suggest that recurrent E-I coupling establishes intrinsic temporal scales for neuronal activity in local circuits. These intrinsic scales are apparent in the resonant properties of the population, which temporally transform excitatory input, selecting components of time-varying input coherent with the resonant oscillation and attenuating non-coherent components.

## RESULTS

### AAV1 and AAV9 transfect excitatory neurons in the cat visual cortex, and constant optogenetic stimulation reveals gamma-band resonance

Recombinant adeno-associated virus (AAV) vectors are widely used as gene-delivery tools (Vasileva and Jessberger, 2005). AAV-mediated expression of ChR2 has been used in several mammalian species, including mice, rats, and non-human primates (Diester et al., 2011; Gerits et al., 2015; Scheyltjens et al., 2015). In this study, three pseudo-typed AAVs, AAV1, AAV5, and AAV9, were applied in the visual cortex of the domestic cat (*Felis catus*). We injected AAVs carrying the gene for hChR2(H134R)-eYFP (eYFP = enhanced yellow fluorescent protein) under the control of the Ca^2+^/calmodulin-dependent protein kinase type II alpha (CaMKIIα) promoter. Injections targeted either area 17, the cat homolog of primate area V1, or area 21a, the cat homolog of primate area V4 (Payne, 1993). All AAV1 and AAV9 injections resulted in robust transfection (which was not the case for AAV5; see STAR Methods). Transfection was evident in confocal fluorescence microscopy (and often in epifluorescence) and in the neuronal responses evoked by light. In total, we transfected neurons in area 17 in four hemispheres of three cats and in area 21a in four hemispheres of four cats.

**Figure 1.**
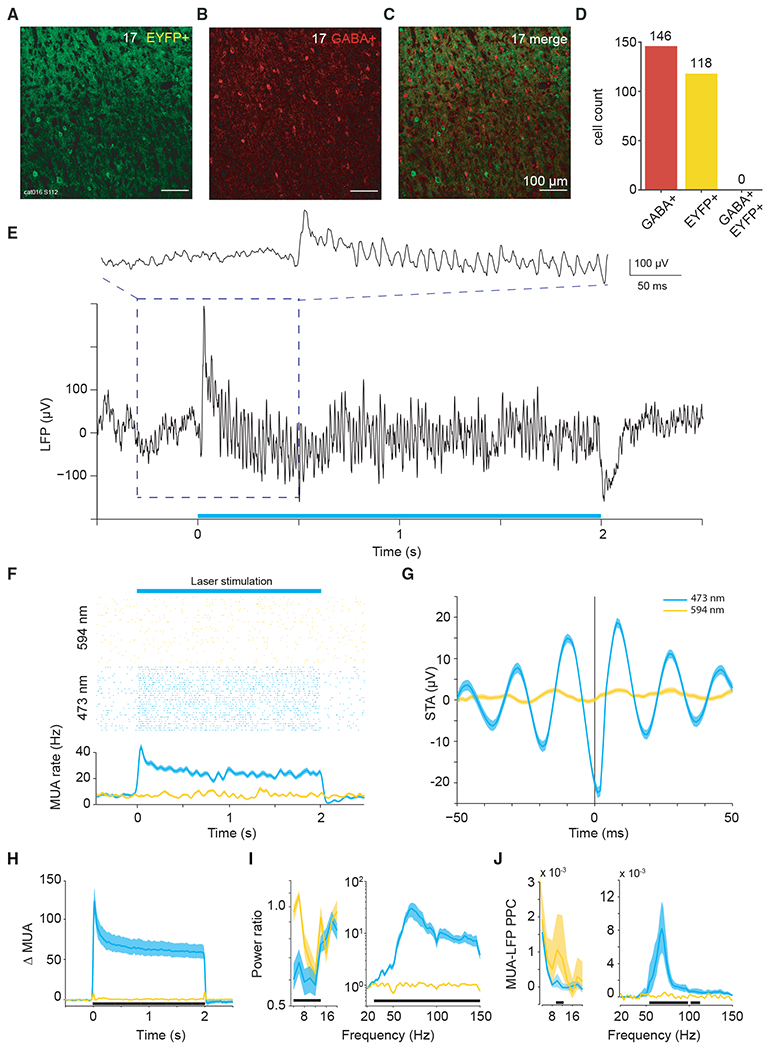
Viral transfection and gamma-band resonance to stimulation (A–C) Confocal microscopy images of immunohistochemistry performed on slices from area 17 after viral transfection. (A) Endogenous fluorescence of ChR2-eYFP. (B) Fluorescence from secondary antibody after staining for GABA^+^. (C) Merged images, testing for neuronal co-labeling with ChR2-eYFP and GABA^+^ antibody. No co-labeled neurons can be found. (D) Counts of GABA^+^-labeled neurons, EYFP^+^-labeled neurons, and co-labeled neurons in area 17. (E) Example recording site in area 17 shows strong gamma-band activity in the local field potential induced by constant illumination. (F) Robust MUA response to constant illumination at the same site. (G) Spike-triggered LFP for example data shown in (E) and (F). (H) Average MUA spike density change as a result of optogenetic stimulation. Smoothed by a Gaussian function (σ = 12.5 ms, truncated at ±2σ). (I) Average LFP power ratio (optogenetic stimulation versus baseline) spectrum. Note different y axis scales for lower and higher frequency ranges. (J) Average MUA-LFP PPC spectrum. Note different y axis scales for lower- and higher-frequency ranges. (I and J) Use ±0.5-s epochs for the analyses from 4 to 20 Hz, and ±0.25-s-long epochs for the analyses from 20 to 150 Hz. (F–J) Blue (yellow) lines show data obtained with 473 (594) nm light stimulation. Shaded area indicates ±1 SEM across trials. (H–J) Black bars at the bottom indicate frequency ranges with statistically significant (p < 0.05) differences between blue and yellow light stimulation, based on a cluster-level permutation test including correction for the multiple comparisons across frequencies.

In two cats, after electrophysiological recordings were completed, brains were histologically processed, and slices were stained for parvalbumin (PV) and/or gamma-aminobutyric acid (GABA) (Figures 1 and S1). One cat had been injected with AAV1-CaMKIIα-hChR2(H134R)-eYFP into area 17. Across several slices and imaging windows of area 17, we identified 264 unequivocally labeled neurons, which showed ChR2-eYFP expression or GABA antibody staining; of those, 146 were positive for GABA and 118 expressed ChR2-eYFP, and there was zero overlap between these groups (Figures 1A–1D). In the same cat, across several additional slices and imaging windows of area 17, we identified 284 unequivocally labeled neurons, which showed ChR2-eYFP expression or PV antibody staining; of those, 145 were positive for PV, and 139 expressed ChR2-eYFP, with four neurons showing clear ChR2-eYFP fluorescence and partial (patchy) PV staining (Figures S1A–S1D). The other cat had been injected with AAV9-CaMKIIα-ChR2-eYFP into area 21a. Across several slices and imaging windows of area 21a, we identified 182 unequivocally labeled neurons, which showed ChR2-eYFP expression or PV antibody staining; of those, 73 were positive for PV, 109 expressed ChR2-eYFP, and there was zero overlap between these groups (Figures S1E–S1H). Thus, ChR2 expression occurred almost exclusively in excitatory neurons.

We performed terminal experiments under general anesthesia 4–6 weeks after virus injection. The transfected portion of cortex was illuminated with blue or yellow laser light (473 or 594 nm), while neuronal spike and local field potential (LFP) activity was recorded. Because ChR2 is a light-activated cation channel, illumination of transfected neurons emulates excitatory synaptic inputs. The external excitatory drive to the network can thus be controlled by modulating the intensity of the illumination. Visual cortex exhibits strong gamma-band synchronization in response to sustained visual stimulation (Gray et al., 1992; Gray and Singer, 1989). Gamma-band synchronization has also been reported during optogenetic activation of excitatory cells in the primary motor cortex of macaque monkeys (Lu et al., 2015), as well as the primary somatosensory cortex and hippocampus of the mouse (Adesnik and Scanziani, 2010; Akam et al., 2012; Stark et al., 2014). We have previously observed gamma-band synchronization in response to constant optogenetic stimulation of excitatory neurons in the visual cortex of the anesthetized cat (Ni et al., 2016). We now present a more detailed analysis of this phenomenon. A single trial of the LFP response to optogenetic stimulation with 2 s of constant blue light from area 17 is shown in Figure 1E. The raw LFP trace reveals strong optogenetically induced gamma that emerged immediately after the onset of stimulation. Figure 1F shows the multi-unit activity (MUA) of this recording site for many interleaved trials of stimulation with blue or yellow light, confirming that activation was selective for blue light. Activation was also specific to regions of cortex expressing ChR2, because laser stimulation with blue or yellow light had no measurable effect for control recordings in the non-transfected cortex (Figures S1I and S1J). Figure 1G shows the spike-triggered average (STA) of the LFP, demonstrating that optogenetic stimulation induced spikes that were locked to the LFP gamma-band component. Results in area 21a were highly similar, and example data are presented in the Supplemental information (Figures S2A–S2C).

This pattern was found very reliably across recording sites. Stimulation with 2 s of constant blue light, as compared with yellow control light, induced strong enhancements in firing rate, which were sustained for the duration of stimulation (Figures 1H and S2D; Wilcoxon rank-sum test = 39,581; p < 0.0001; n = 163 sites in 4 cats). The ratio of LFP power during stimulation versus pre-stimulation baseline showed an optogenetically induced gamma-band peak around 70 Hz (Figures 1I and S2E; Wilcoxon rank-sum test = 14751; p < 0.0001; n = 99 sites in 4 cats). Note that the gamma-band peak frequency varied across animals and recording sessions, as shown previously (Ni et al., 2016). The LFP gamma-power changes reflected changes in neuronal synchronization, because optogenetic stimulation also induced strong MUA-LFP locking in the gamma band, as quantified by the MUA-LFP PPC (Figures 1J and S2F; PPC = pairwise phase consistency; Wilcoxon rank-sum test = 9,389; p < 0.0001; n = 84 sites in 4 cats). In addition to the induction of gamma-band synchronization, optogenetic stimulation also reduced LFP power at 4–14 Hz (Figures 1I and S2E, inset) and MUA-LFP locking at 10–12 Hz (Figures 1J and S2F, inset). These reductions of lower-frequency synchronization are reminiscent of effects of visual stimulation and/or selective attention in awake macaque area V4 (Fries et al., 2008b; Mitchell et al., 2009).

### Greater excitation increases magnitude and frequency of resonance

**Figure 2.**
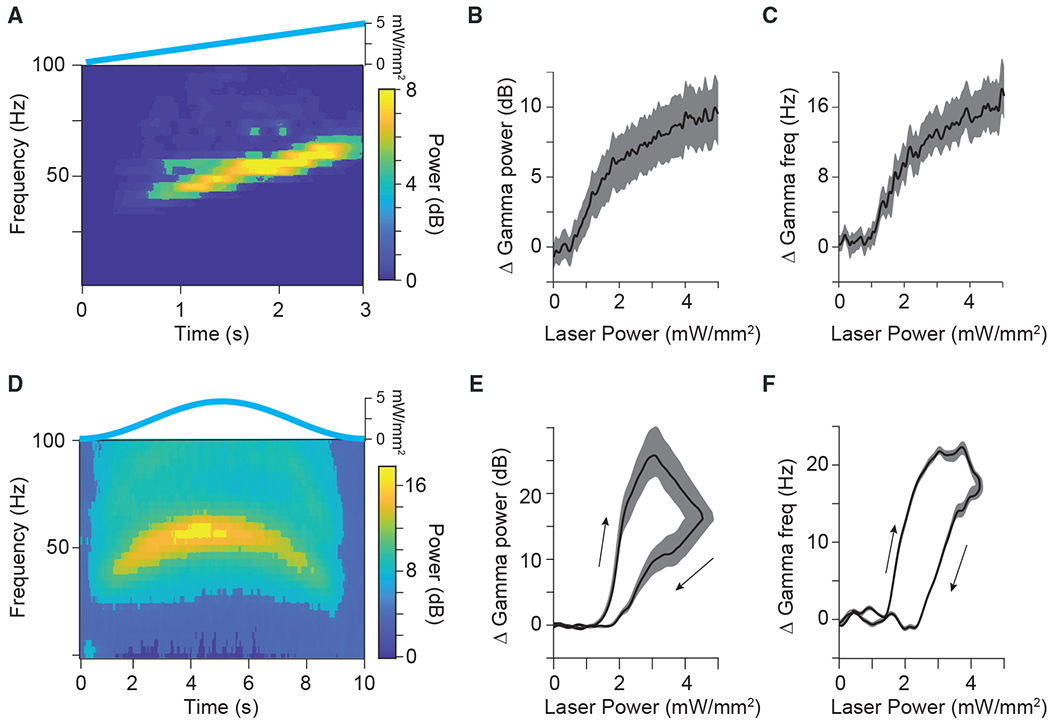
Bandwidth and hysteresis of gamma-band resonance (A) Time-frequency plot for an example site in area 21a in response to a slowly increasing ramp stimulus, shown on top. (B) Group result for ramp stimulation shows that the power of the gamma-band resonance increases sublinearly with increasing excitatory drive (n = 58 sites in 5 cats). (C) Same as in (B), but for the frequency of the gamma-band resonance. (D) Time-frequency plot for an example site in area 21a to a slow Gaussian temporal profile, shown on top. (E) Group results showing the change in power of gamma-band resonance as a function of laser intensity during slow Gaussian stimulation (n = 52 sites in 5 cats). (F) Same as in (E), but for frequency of gamma-band resonance. Arrows in (E, F) indicate hysteresis in response to increasing (upper arrow) versus decreasing (lower arrow) laser power. Shaded areas in (B), (C), (E), and (F) indicate ±1 SEM across recording sites.

We next characterized the bandwidth of the network resonance by varying the excitation in the local network. Models and empirical data have both suggested that the frequency of gamma oscillations can increase with increasing excitation (Jia et al., 2013; Lowet et al., 2017; Ray and Maunsell, 2010; Roberts et al., 2013; Traub et al., 1996). We therefore slowly increased excitation linearly in time (ramp stimulation, 3 s) to generate increasing excitation in the local network. A time-frequency plot for an example recording site in area 21a is presented in Figure 2A. We found that the network resonance varied non-linearly with the input excitation. Rather than scaling linearly with light strength, network resonance began only after a critical level of excitation was reached (Figures 2A and 2B), as previously established *in vitro* and in models (Börgers et al., 2005; Traub et al., 1996). Power and frequency increased sub-linearly with increasing excitation (Figures 2B and 2C). Interestingly, previous studies reported that optogenetic drive of excitatory cells in the somatosensory cortex and hippocampus of the mouse with light ramps resulted in gamma-band synchronization with a constant frequency (Adesnik and Scanziani, 2010; Akam et al., 2012), and increasing the slope of the ramp gave rise to higher-frequency synchronization in the somatosensory cortex (Adesnik and Scanziani, 2010). To further investigate additional non-linearities in the resonance, we stimulated the network with slow symmetric excitation profiles (single-slow-sine-wave stimulation, 10 s). A time-frequency plot for an example recording site in area 21a is presented in Figure 2D. Single-slow-sine-wave stimulation revealed amplitude and frequency hysteresis, with the amplitude and frequency of the network resonance increasing sub-linearly after a critical point of excitation was reached and slowing down more quickly upon waning excitation (Figures 2E and 2F).

### Models reveal the potential role of non-inactivating M-current in hysteresis

To investigate the network mechanisms underlying the observed resonance phenomena and the hysteresis, we constructed mathematical models of recurrently coupled E-I neurons. To this end, we used a well-established biophysically realistic pyramidal-interneuron network (PING) model (Börgers, 2017), without additional tuning. We initially investigated a model composed of two populations of single-compartment neurons implementing Hodgkin-Huxley dynamics. The excitatory population is based on a simplified model of pyramidal cells (Traub et al., 1991), and the inhibitory population is based on a simplified model of PV^+^ basket cells (Wang and Buzsáki, 1996). The network has a synaptic model that permits a gradual rise of synaptic gating (Wang, 1999). This model produced strong gamma-band synchronization, as has been reported extensively (Börgers and Kopell, 2003; Figures S3A and S3B).

The PING network reproduced the experimentally observed increase in the power and frequency of the resonance with increased external drive (Figures S3C and S3D). Such increases have also been described *in vitro* (Traub et al., 1996) and in simple networks (Wilson and Cowan, 1972). We implemented a simple LIF network and found that it also exhibited power and frequency increases with increased excitatory drive (Figures S4A and S4B). However, neither the PING nor the LIF model were able to reproduce the experimentally observed hysteresis effects (Figures S3C, S3D, S4A, and S4B). We therefore modified the PING model by adding a non-inactivating M-current to the excitatory population (PING+M model). The M-current is a potassium current that is active at rest and during depolarization and raises the threshold for action potential generation. The PING+M model has lower firing rates and a lower resonant frequency for equal excitatory drive, as compared with the PING model (Figures S3E and S3F). The PING+M model was able to produce both power and frequency hysteresis in qualitative concordance with our experimental findings (Figures S3G and S3H, as compared with Figures 2E and 2F). The hysteresis evident in the PING+M model was considerably less pronounced than what was observed experimentally, suggesting that more factors, such as additional currents, or cell classes, are likely to contribute to the hysteresis observed *in vivo*.

### Rhythmic input that matches resonance is preferentially transmitted

**Figure 3.**
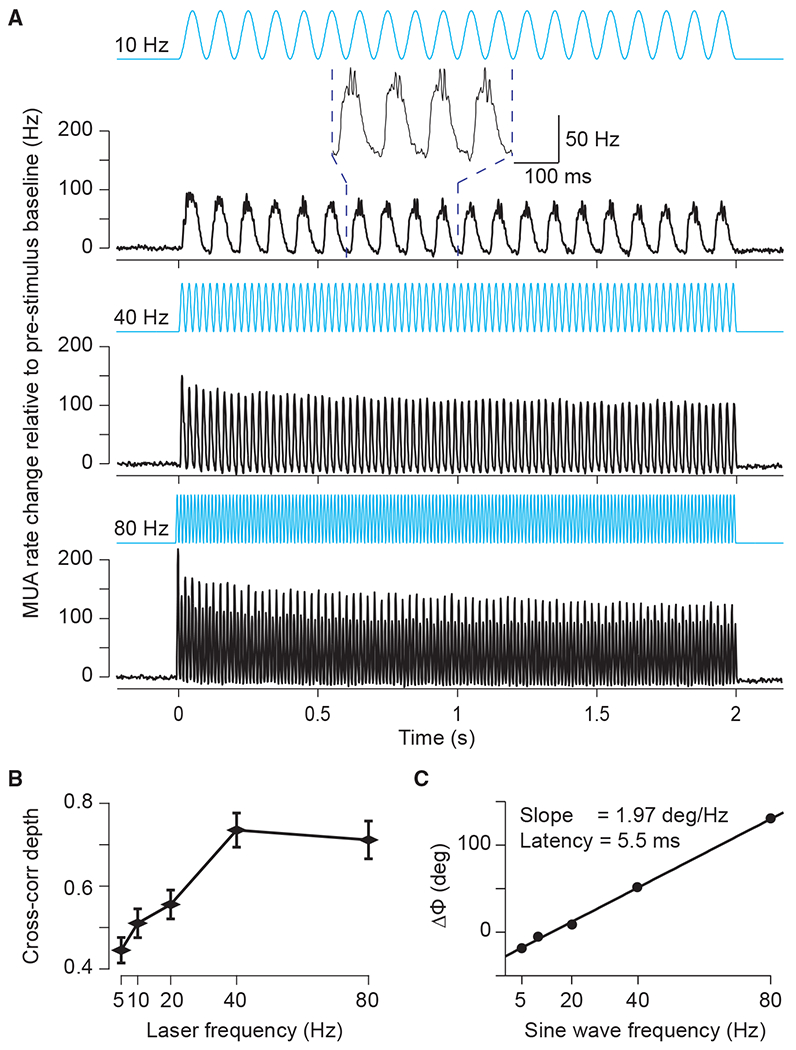
MUA responses to sinusoidal stimulation (A) MUA spike density (Gaussian smoothing with σ = 1.25 ms and truncated at ±2σ) for 10- (top), 40- (middle), and 80-Hz (bottom) sinusoidal stimulation, respectively. The inset shows an enlarged version of a few cycles to illustrate the gamma-band resonance induced at the peak of the depolarizing phase of the 10-Hz sinusoid. Data were baseline subtracted (−0.5 to 0 s) and averaged over all MUA recording sites (n = 60 in 4 cats). Error regions for ±1 SEM across recording sites are smaller than line width. (B) Modulation depth quantified as peak-to-trough distance of the Pearson cross-correlation coefficient as function of the frequency of stimulation. (C) Peak latency from stimulation to MUA response as a function of frequency. The text inset gives the slope and the corresponding latency between optogenetic stimulation and neuronal response.

We next returned to the empirical data and sought to investigate whether the output of the local network, assessed by spike output, demonstrates a preference for temporally varying inputs with a timescale matching the network resonance, as has been suggested by computational models (Sherfey et al., 2018). We drove rhythmic excitation in the network with sinusoidal stimulation of 5, 10, 20, 40, and 80 Hz. Light intensity was adjusted per recording site (see STAR Methods) and was kept constant for a given site across the different stimulation frequencies. Sinusoids of all applied frequencies resulted in clear increases in firing rate, with strong rhythmicity at the stimulation frequency (Figure 3). We calculated spike density functions, subtracted the baseline values, and averaged them across recordings sites. Figure 3A shows those average spike densities for 10, 40, and 80 Hz. Note that 10-Hz stimulation resulted in not only an entrained 10-Hz response but also bursts of gamma-band synchronization around the peak of excitatory drive, in agreement with a previous report in rodent hippocampus (Butler et al., 2016). Note also that 80-Hz stimulation did not result in simple entrainment to the 80-Hz stimulation, but that the response varies on alternate cycles, exhibiting a prominent sub-harmonic to the driving frequency at 40 Hz that was stable for the entire 2-s stimulation period.

To capture entrainment by the optogenetic stimulation, we calculated the Pearson cross-correlation coefficient between the respective sinusoid and the resulting spike density as a function of time lag between the two (Figures 3B and S5A). We quantified the strength of entrainment as the peak-to-trough distance of the cross-correlation functions (Figure 3B). Sinusoidal stimulation resulted in entrainment that increased with stimulation frequency to peak at 40 Hz and weakly decreased at 80 Hz (oneway ANOVA, p = 1.6E–9, *F*_(4,295)_ = 11.25). The bandwidth of the preferential entrainment matches well the bandwidth found by varying excitation with ramps and Gaussian stimulation, and the small fall-off at frequencies above the network resonance is in good agreement with previous modeling work (Sherfey et al., 2018).

Sinusoidal stimulation of different frequencies enabled estimation of neuronal response latencies by computing the slope of relative phases between the stimulation signal and the output MUA across stimulation frequencies (Figures S5B–S5F; see STAR Methods for an expanded discussion of this method). Figure 3C presents the relative-phase spectrum and reveals a strictly linear relationship, a signature of a fixed time lag. The slope of this linear relationship indicates a latency of 5.5 ms, in good agreement with previous reports (Boyden et al., 2005; Cardin et al., 2009).

### Optogenetic white-noise stimulation reveals a causal role of gamma

Finally, and crucially, we emulated input with a white-noise characteristic. White noise realizes continuously unpredictable values (innovation) and thus shows no autocorrelation, i.e., no correlation with its own past or future. Therefore, time-lagged correlations between the optogenetically emulated neuronal input and the neuronal spike output cannot be caused by time-lagged correlation within the input but can be unequivocally attributed to a time-lagged correlation between input and output. A time-lagged correlation between an experimentally controlled input and the observed spike output provides direct evidence for a causal role of the input. Importantly, white-noise stimulation enabled us to determine the causal roles separately for each frequency of the spectrum. That is, white-noise excitatory drive during recording of spike output allowed us to determine the directed transfer function of the observed network.

**Figure 4.**
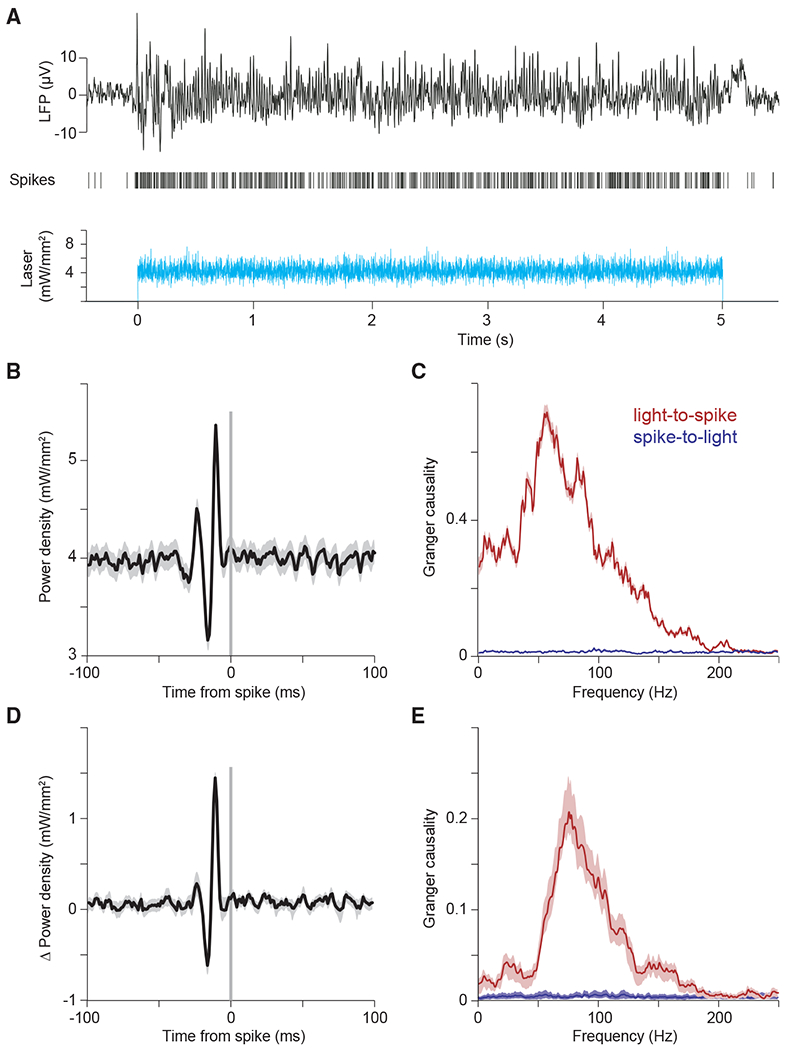
The component of white-noise stimulation coherent with network resonance is transmitted as MUA (A–C) Example single-trial LFP and MUA response to optogenetic white-noise stimulation. The bottom panel shows the white-noise time course of laser intensity. The sequence of vertical lines above it indicates time points of MUA spike occurrence. The black continuous line on top shows the LFP. (B) Spike-triggered average (STA) of laser power density, triggered by the spikes recorded at one example recording site. (C) Granger causality (GC) spectrum for the data shown in (B). Red line shows GC from light to spikes; blue line shows GC from spikes to light (as control). (D and E) Same as (B) and (C), but for the average across recording sites (n = 13 sites in 3 cats).

We employed optogenetic stimulation with light intensities following a Gaussian random process (sampled at ≈ 1,000 Hz) with a flat power spectrum (Figure 4A, bottom trace). This white-noise stimulus contains the same energy at all frequencies up to 500 Hz. Light intensities were titrated such that firing rates were in the lower half of the dynamic range of the recorded neurons in response to optogenetic stimulation. Figure 4A shows an example LFP and MUA recording for an example trial of white-noise stimulation.

To reveal the temporal input patterns most reliably driving spikes, we aligned the white-noise time series that drove the laser to the spikes and averaged it. Figure 4B shows the resulting STA light power density for an example recording site. We found that spikes were preceded by a characteristic sequence of increased and decreased light intensity, with a peak-to-peak cycle length corresponding to 75 Hz, suggesting a causal role of the gamma band in eliciting spikes. To quantify this causal influence in a frequency-resolved manner, we calculated the Granger causality of the time-varying light intensity onto the spike train. This revealed a clear peak in the gamma band (Figure 4C, red). As a control, we also calculated the Granger causality of the spike train onto the light, which confirmed values close to zero, as expected (Figure 4C, blue). We found very similar effects in the average over recording sites (Figures 4D and 4E; n = 13 sites in 3 cats), confirming a predominant role of the gamma band in causing spikes.

### Models reveal key role of feedback inhibition in transmission of coherent input

**Figure 5.**
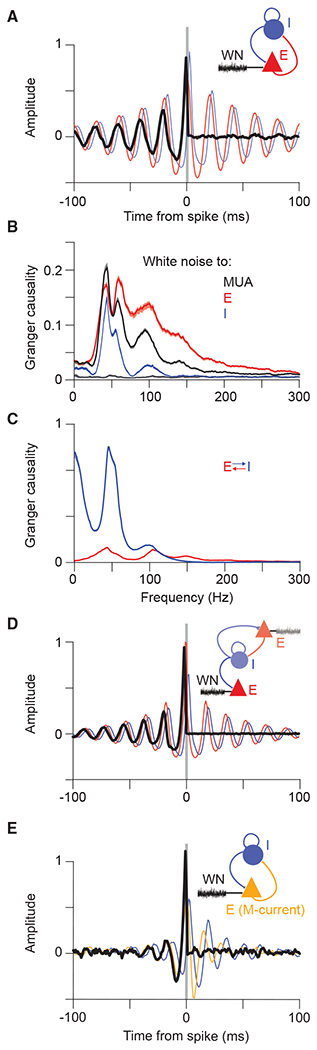
Computational modeling reveals potential mechanism underlying preferential transmission of coherent input (A) Spike-triggered average of white-noise input signal (black), network excitation (red), and inhibition (blue) demonstrates preferential transmission of gamma-frequency input that matches the intrinsic dynamics of the network. White-noise averaging was triggered by spikes of all excitatory neurons; results for inhibitory neurons or all neurons (total MUA) are shown in Figure S8C. Inset depicts a schematic of the PING model. (B) GC spectrum from white-noise input to total MUA (black), excitatory spikes (red), and inhibitory spikes (blue). Spectra from MUA and spikes to white noise are presented in muted color and overlap near zero. (C) GC spectra between excitation and inhibition in the network. Spectrum from excitatory spikes to inhibitory spikes (blue) and vice versa (red). (D) Spike-triggered averages in the model with two excitatory populations. Averages display the spike-triggered white noise (black) driving the second excitatory population and illustrate entrainment by the excitation (red) and inhibition (blue) of the recurrently coupled PING network. Inset depicts a schematic of the model. The PING network shown on top, in lighter colors, contains a first excitatory population and an inhibitory population and generates gamma upon white-noise input. The resulting rhythmic inhibition is fed into a second excitatory population, shown on the bottom, which is driven by independent white noise. (E) Spike-triggered averages as in (B), but for the PING+M model. Inset depicts a schematic of PING+M model driven by white noise. E, excitatory neuron pool; I, inhibitory neuron pool; WN, white-noise input.

In order to better understand the network behavior under external drive with temporal white noise, we first returned to the PING model without M-current (Figure 5A, inset). When we stimulated this model with white noise, we found the same signature of frequency-dependent transmission as in our experiments (Figures 5A–5C, as compared with Figures 4B–4E). This effect was also evident in the LIF network (Figure S8A) and in a PING network without I-to-I connectivity (Figure S8B). The model permitted us to separate excitatory from inhibitory activity, and we found that input in-phase with network excitation and phase-advanced with respect to network inhibition is preferentially transmitted (Figure 5A). We computed the Granger causality spectra between the white-noise input and the MUA in the network and found a high degree of qualitative similarity to our empirical spectra (Figure 5B, black line, and Figures 4C and 4E). Again, because we could separate excitation from inhibition in the model, we could separately investigate the transfer from the white-noise input to the excitatory (Figure 5B, red line) and the inhibitory (Figure 5B, blue line) activity of the network (see Figure S8C for corresponding STAs). This suggests that the white-noise components transmitted to network excitation are broader, as compared with the components transmitted to the inhibition. We further investigated the transfer between the E-I units of the network (Figure 5C). Excitatory units transmitted variance at gamma, and additionally significant low-frequency variance, to the output of the inhibitory network, whereas inhibitory units transmitted primarily gamma-band components back to the excitatory units (Figure 5C).

To further understand the mechanisms of preferential transfer, we next asked whether the excitatory population receiving the white-noise innovation must project to the inhibitory population and thereby entrain a rhythm, or whether a resonant pool, isolated from the white-noise innovation, but projecting inhibitory synapses to that population, could implement selective transmission. We simulated a network with one population of inhibitory neurons and two separate populations of excitatory neurons (Figure 5D, inset). A first excitatory population (illustrated at the top of the inset in Figure 5D) was recurrently connected to the inhibitory population, and when this circuit was driven by white-noise input, it generated gamma resonance. The resulting output of the inhibitory population was fed into the second excitatory population (illustrated at the bottom of the Figure 5D, inset), which did not project back to the inhibitory pool. White-noise input to this second excitatory population was preferentially transmitted, if it was coherent with and phase-advanced to the gamma-rhythmic inhibition (Figure 5D). Thus, rhythmic gating can be exerted by one circuit onto a separate, gated circuit.

Finally, because the M-current had been necessary to explain the experimentally observed hysteresis effects, we asked whether selective transmission occurs in the PING+M model (Figure 5E, inset; Figure S7). Using the same model parameters as used for the investigation of hysteresis, we performed analysis of the network under white-noise stimulation. We found that the PING+M model also exhibited selective transmission (Figure 5E). Intriguingly, the M-current significantly reduced the timescale of the selective transmission, producing spike-triggered white noise in better agreement with that found experimentally (Figure 5E as compared with Figures 4B and 4D). Together with the hysteresis results, the close qualitative match between the experimental and the PING+M spike-triggered white-noise results suggests the influence of some form of spike-frequency adaptation. As mentioned above, it is likely that additional cell classes, or conductances, may play a role *in vivo* and require further investigation. In any case, given the potential role of the M-current suggested by our findings, it would be interesting to investigate the impact of acetylcholine on the phenomena described here. Acetylcholine can have an antagonistic effect on the M-current via muscarinic receptors, and in the PING+M model this would increase the power and frequency of the circuit resonance, enhance the amplitude and timescale of selective transmission, and reduce the hysteresis of the gamma-band resonance. Intriguingly, a number of previous studies in the cat visual cortex have already described increased gamma-band synchronization after electrical stimulation of the midbrain reticular formation (Munk et al., 1996), which appears to depend on muscarinic receptors (Rodriguez et al., 2004).

## DISCUSSION

Visual stimulation induces clear gamma-band synchronization in the cat visual cortex, both during wakefulness (Fries et al., 2002; Gray and Viana Di Prisco, 1997) and anesthesia (Gray et al., 1992). We recorded LFPs and neuronal spike output in the visual cortex of anesthetized cats, while optogenetically emulating external, excitatory inputs to pyramidal neurons with precise experimental control. Controlling external excitatory drive allowed us to investigate the functional consequences of the cortical gamma-band resonance. Optogenetic excitation with a variety of temporal patterns produced gamma-band activity qualitatively similar to that found for visual stimulation. A better understanding of cortical resonance sheds light on the dynamic transformations performed by the local circuit and reveals how time-varying excitation is transmitted.

We confirmed that the visual cortex transforms constant excitation into strong gamma-band synchronization, producing rhythmic spike output similar to visual stimulation (Ni et al., 2016). Slowly increasing excitation with ramps increased the strength and frequency of synchronization and revealed a threshold of excitation necessary for the ignition of synchronization. A positive correlation between excitatory drive and the strength and frequency of gamma-band synchronization has been predicted by computational models, demonstrated *in vitro,* and is reminiscent of effects seen *in vivo* for visual contrast and salience (Fries, 2015; Hadjipapas et al., 2015; Jia et al., 2013; Lowet et al., 2017; Ray and Maunsell, 2010; Roberts et al., 2013; Traub et al., 1996). Slow, temporally symmetric excitation profiles demonstrated profound hysteresis in both the strength and the frequency of the synchronization. Although hysteresis in synchronization has so far been unreported to our knowledge, it is reminiscent of effects seen when visual contrast is symmetrically varied (for example, see figure 3 of Ray and Maunsell, 2010). Modeling indicated that hysteresis could arise from spike-frequency adaptation via a non-inactivating potassium current (M-current), suggesting that acetylcholine effects on the M-current may modify the dynamics of gamma-band resonance (Börgers et al., 2005; Fellous and Sejnowski, 2000; Fisahn et al., 1998; Munk et al., 1996; Rodriguez et al., 2004). The observed hysteresis could play a powerful role in differentiating populations of cells with increasing versus decreasing excitation, even if the total level of excitation in the populations is equal. Future studies should elucidate the rich, non-linear features of the resonance described here, such as the minimal excitatory drive required for resonance, its dynamic range, and its interaction with neuromodulatory signals.

Varying external drive on faster timescales enabled us to investigate how cortical resonance selectively transmits components of dynamic input. The effect of the network resonance on variable input was first demonstrated for rhythmic, sinusoidal excitation. Sinusoidal drive was transformed by the network into spike output with a fidelity that increased up to 40 Hz and declined slightly for 80 Hz. Intriguingly, slow sinusoidal input gave rise to bursts of gamma-band synchronization at the peaks.

Crucially, the precise temporal control afforded by optogenetics enabled characterization of the network response to stochastic, white-noise sequences. White-noise stimulation facilitated causal analysis of network transmission: from external excitatory input to neuronal spike output. The gamma-band component of the stochastic input preferentially drove spiking in the neuronal population. Thus, feline visual cortex is predisposed to transform external excitation with a variety of temporal profiles into gamma-rhythmic spike output. Further, the resulting gamma-rhythmic output is ideally suited to preferentially drive activity in downstream populations.

Network resonance emerges from the interaction between E-I elements. In computational models, including those presented here, network resonance is determined largely by feedback inhibition (Börgers and Kopell, 2003; Buzsáki and Wang, 2012; Tiesinga and Sejnowski, 2009; Whittington and Traub, 2003). Although resonance arises in reduced models with homogeneous cellular properties, the cat visual cortex contains a great deal of heterogeneity. The dominant gamma-band resonance we observed could be caused by intracellular mechanisms, network properties, or combinations of both. Intracellular transfer functions have been characterized for assorted cell classes using *in vitro* electrophysiology and optogenetics. Although there is diversity depending on morphology and channel composition, the dominant cell class we drove with light, pyramidal cells, typically exhibits a low-pass characteristic. Previous work characterized the transfer function of a variety of opsins, including the opsin used here (hChR2(H134R)), in cultured pyramidal cells and found that transfer peaked at 3 Hz and declined smoothly for higher frequencies, with currents reduced by half at ~40 Hz (ChR2R in figure 1 of Tchumatchenko et al., 2013). Therefore, the gamma-band resonance observed in the present study is most likely not due to the opsin or electrical properties of the individual neurons, but rather predominantly determined by feedback inhibition in the network (Buzsáki and Wang, 2012). This network mechanism is likely assisted and amplified by cellular mechanisms. Interneurons can show 1:1 phase locking to suprathreshold sinusoidal current injections up to 50 Hz (Fellous et al., 2001). When the transfer function from injected current to spike times is directly measured for cortical interneurons in slices of ferret prefrontal cortex, it reveals a broad peak in the gamma range (Hasenstaub et al., 2005). Additionally, specialized classes of excitatory neurons have been described in cat and macaque visual cortex, with properties that likely promote gamma-band resonance (Gray and McCormick, 1996; Onorato et al., 2020).

Interestingly, the STA revealed that spikes were preceded not only by rhythmic peaks but also by rhythmic troughs, suggesting that input that matches the intrinsic timescale of feedback inhibition is preferentially transmitted. In a driven state, network excitation and inhibition wax and wane with a delay determined by features of synaptic connectivity. This creates windows of enhanced susceptibility to the external drive, and the pace of network inhibition will preferentially permit excitatory cells to transmit components of their time-varying extrinsic drive that match the endogenous dynamics (Fries, 2015). Exogenously driven excitatory spikes will subsequently drive inhibitory neurons and renew the cycle of feedback inhibition. If excitation arrives out of phase with the network rhythm, it can prematurely drive inhibition in a feedforward manner, and sufficient premature capture of inhibition will lead to desynchronization of the inhibitory pool. Such premature forcing is kept in check by the strong synchronization within the inhibitory pool, via dense I-I coupling. Thus, exogenous excitation competes with the endogenous pace set by strong feedback inhibition.

STA analysis has been used to characterize the input-output relationship of single neurons, both in terms of their receptive field properties (Chichilnisky, 2001; Pillow et al., 2008) and their resonance properties (Bryant and Segundo, 1976; Mainen and Sejnowski, 1995; Marmarelis and Naka, 1972). It is also routinely used to estimate the locking of neurons to simultaneous population activity, either by spike-triggered LFP averaging (Fries et al., 1997) or spike-triggered covariance analysis (Pillow et al., 2008). STA analysis of both intracellularly recorded membrane potentials (Azouz and Gray, 2008; Hasenstaub et al., 2005) and LFPs (Fries et al., 1997) has revealed strong gamma-band phase-locking during visual stimulation. As membrane potentials and LFPs reflect synaptic currents (Pesaran et al., 2018), these observations are consistent with a scenario in which spikes are specifically caused by the gamma component of synaptic inputs. However, these findings are also consistent with a scenario in which visual stimulation induced gamma-rhythmic neuronal activity reflected in both spiking and LFP, without a specific causal role of gamma-rhythmic inputs. Optogenetic white-noise stimulation allowed us to isolate the effect of external gamma-rhythmic drive from ongoing synchronization. We were therefore able to demonstrate the causal role of network resonance in selectively transmitting the gamma component of time-varying external input. Importantly, the gamma-rhythmic component of the spike-triggered white-noise average cannot be explained by the mere fact that the stimulation induced gamma-rhythmic neuronal spiking. Rather, it required that spikes were time locked (and thereby phase locked) to the relevant temporal pattern in the white noise. If white noise had simply induced spikes that were gamma rhythmic, but not phase locked, to the gamma component of the white noise, the STA of the white noise would have been flat. However, the STA revealed significant modulation in the gamma band, suggesting that spikes were preferentially driven by the input’s gamma components.

The gamma synchronization produced by white-noise input was weaker and more unstable than that produced with constant stimulation (Figure S6). During constant stimulation, the exogenous drive lacks temporal structure, and network dynamics are dominated by the endogenous resonance. However, during white-noise stimulation, endogenous dynamics are perturbed by broadband exogenous drive, resulting in irregular, fragmented synchronization. Similarly, gamma-band activity in macaque V1 is strong when induced by a smoothly moving grating and substantially reduced by the addition of random motion (Kruse and Eckhorn, 1996). Interestingly, temporally variable exogenous drive leads to precise spike timing, increased stimulus information, and improved perceptual discrimination (Buracas et al., 1998; Christensen et al., 2019; Mainen and Sejnowski, 1995). Complementary results suggest that endogenous gamma dynamics provide additional temporal structure that can enhance the information communicated by neurons (Azouz and Gray, 2003; Harris et al., 2003; Womelsdorf et al., 2012). Together, these results suggest that networks balance the deviations introduced by exogenous drive with the timescale imposed by their endogenous dynamics. Indeed, exogenous transients may function as an external clock to synchronize activity and facilitate transmission, while under continuous or slowly varying drive, resonance may assume the role of timekeeper and discretize transmission into synchronous packages so as to maximize their effect on downstream populations. Under such a regimen, temporal information imposed by a variable stimulus will be faithfully conveyed, and in the absence of exogenous temporal structure, the synchronization imposed by network resonance will endow neuronal communication with increased reliability and precision (Fries, 2015). The balance of exogenous and endogenous drive is likely to fluctuate dynamically according to their relative strength or other variables that can alter the dynamic set point of the circuit. The flexible balancing of extrinsic and intrinsic factors provides a powerful means to selectively amplify and propagate or suppress and gate sensory signals according to the behavioral state or goals.

The experiments reported here were limited to the visual cortex and have focused on the gamma-band resonance prominent in the activated visual cortex (Brunet et al., 2015; Gray and Singer, 1989; Onorato et al., 2020). However, all recurrently coupled excitatory-inhibitory networks are likely to demonstrate similar resonances, which will function to selectively filter their input and temporally tune their output. This reasoning predicts that spikes in other areas, in which other rhythms predominate (Brown et al., 1998; Csicsvari et al., 2003; Fries, 2009; Gregoriou et al., 2009; Pesaran et al., 2002), might be caused predominantly by the corresponding rhythm in their input. Likewise, because our experiments were carried out in anesthetized animals, we could not establish the behavioral relevance of the reported phenomena. Previous work has used white-noise flicker to investigate the reverberatory nature of visual responses (VanRullen and Macdonald, 2012) and attentional gating of stimulus information (Grothe et al., 2018). These promising results suggest that optogenetic stimulation in behaviorally engaged circuits may provide a powerful means to probe the dynamic routing of information between relevant brain areas.

The filtering and preferential transmission reported here suggest that resonance is a compelling mechanism by which to achieve flexible communication (Izhikevich et al., 2003). The resonant frequency of a circuit or population will determine the communication channel of that circuit, and coherent input will be transmitted, whereas non-coherent input is suppressed (Akam and Kullmann, 2010). Indeed, distinct resonances are likely to exist within a single cortical area, for example, between distinct neuronal subpopulations, projections, or laminae. For example, superficial and deep layers in macaque areas V1, V2, and V4 show very different rhythms during activation. Although superficial layers express strong gamma synchronization, deep layers show an alpha-beta rhythm (Buffalo et al., 2011; van Kerkoerle et al., 2014). Rhythms can also change dynamically depending on intrinsic or extrinsic factors, such as behavioral state or cognitive context, and such changes might alter resonances and input-output functions, perhaps via modulatory signals (Gulbinaite et al., 2019). A hierarchy of areas with intrinsic resonances could act to selectively distinguish and propagate feedforward and feedback signals in the spectral domain, as has been suggested by functional-anatomical studies (Bastos et al., 2015; Michalareas et al., 2016; van Kerkoerle et al., 2014) and modeling (Lee et al., 2013). It will be a highly interesting task for future studies to probe resonances in different areas, layers, projections, or cell classes and especially in different cognitive contexts. Note that the approach presented here can also be used to investigate the transfer between input to one neuronal group and the spike output of another neuronal group, with the two groups possibly residing in different layers and/or areas. With recordings at site A and stimulation at sites B and C, it might be possible to characterize not only the spectral transfer function from B to A but also the frequency-resolved modulatory influence of C on this transfer function. By facilitating such investigations, the presented approach provides a novel framework in which to study the mechanisms underlying flexible neuronal communication.

## STAR★METHODS

### RESOURCE AVAILABILITY

#### Lead contact

Further information and requests for resources and reagents should be directed to and will be fulfilled by the Lead Contact, Pascal Fries (pascal.fries@esi-frankfurt.de).

#### Materials availability

This study did not generate new unique reagents.

#### Data and code availability

The datasets and code supporting the current study are available from the corresponding authors on request.

### EXPERIMENTAL MODEL AND SUBJECT DETAILS

Eight adult domestic cats (*felis catus;* four females; mean age 4.2 years; range 3-8 years) were used in this study. We used cats because the physiology with regard to gamma is highly similar to human and non-human primates (Fries et al., 2008a), both during wakefulness (Fries et al., 2002; Gray and Viana Di Prisco, 1997) and light anesthesia (Gray et al., 1992). Data from the same animals were used in a previous study (Ni et al., 2016). All procedures complied with the German law for the protection of animals and were approved by the regional authority (Regierungspräsidium Darmstadt). After an initial surgery for the injection of viral vectors and a 4-6 week period for opsin expression, recordings were obtained during a terminal experiment under general anesthesia.

### METHOD DETAILS

#### Viral vector injection

For the injection surgery, anesthesia was induced by intramuscular injection of ketamine (10 mg/kg) and dexmedetomidine (0.02 mg/kg), cats were intubated, and anesthesia was maintained with N_2_O:O_2_ (60/40%), isoflurane (~1.5%) and remifentanil (0.3 μg/kg/min). Four cats were injected in area 17 and another four cats in area 21a. Rectangular craniotomies were made over the respective areas (Area 17: AP [0, −7.5] mm; ML: [0, 5] mm; area 21a: AP [0,−8] mm, ML [9, 15] mm). The areas were identified by the pattern of sulci and gyri, and the dura mater was removed over part of the respective areas. Three to four injection sites were chosen, avoiding blood vessels, with horizontal distances between injection sites of at least 1 mm. At each site, a Hamilton syringe (34 G needle size; World Precision Instruments) was inserted with the use of a micromanipulator and under visual inspection to a cortical depth of 1 mm below the pia mater. Subsequently, 2 μl of viral vector dispersion was injected at a rate of 150 nl/min. After each injection, the needle was left in place for 10 min before withdrawal, to avoid reflux. Upon completion of injections, the dura opening was covered with silicone foil and a thin layer of silicone gel, the trepanation was filled with dental acrylic, and the scalp was sutured.

We first tried to transfect with AAV5, because this serotype had been successfully used in many studies on different species (Diester et al., 2011). In one cat, area 17 of the left hemisphere was injected with AAV5-CamKIIα-ChR2-eYFP (titer 4*10^13^ GC/ml). However, this did not result in detectable ChR2-eYFP expression. This failure of AAV5 expression is consistent with one previous study suggesting that AAV5 is not able to provide transduction in the cerebral cortex of the cat (Vite et al., 2003). Subsequently, we tried both AAV1 and AAV9 and found robust transfection with both of these serotypes. In one cat, area 17 in the left hemisphere was injected with AAV1-CamKIIα-hChR2(H134R)-eYFP (titer 8.97*10^12^ GC/ml) and area 17 in the right hemisphere with AAV9-CamKIIα-ChR2-eYFP (titer 1.06*10^13^ GC/ml). In two cats, area 17 of the left hemisphere was injected with AAV1-CamKIIα-hChR2(H134R)-eYFP (titer: 1.22*10^13^ GC/ml). In four cats, area 21a of the left hemisphere was injected with AAV9-CamKIIα-hChR2(H134R)-eYFP (titer: 1.06*10^13^ GC/ml). The DNA plasmids were provided by Dr. Karl Deisseroth (Stanford University, Stanford, CA). AAV5 viral vectors were obtained from UNC Vector Core (UNC School of Medicine, University of North Carolina, USA); AAV1 and AAV9 viral vectors were obtained from Penn Vector Core (Perelman School of Medicine, University of Pennsylvania, USA).

#### Neurophysiological recordings

For the recording experiment, anesthesia was induced and initially maintained as during the injection surgery, only replacing intubation with tracheotomy and remifentanyl with sufentanil. After surgery, during recordings, isoflurane concentration was lowered to 0.6%–1.0%, eye lid closure reflex was tested to verify narcosis, and vecuronium (0.25mg/kg/h i.v.) was added for paralysis during recordings. Throughout surgery and recordings, Ringer solution plus 10% glucose was given (20 ml/h during surgery; 7 ml/h during recordings), and vital parameters were monitored (ECG, body temperature, expiratory gases).

Each recording experiment consisted of multiple sessions. For each session, we inserted either single or multiple tungsten microelectrodes (~1 MΩ at 1 kHz; FHC), or three to four 32-contact probes (100 μm inter-contact spacing, ~1 MΩ at 1 kHz; NeuroNexus or ATLAS Neuroengineering). In one cat, one 16-contact probe with 150 μm inter-contact spacing and one 46 μm optic fiber, and one 16-contact probe with 150 μm inter-contact spacing and four 46 μm optic fibers were used (Plexon V- and U-probe, respectively). Standard electrophysiological techniques (Tucker Davis Technologies, TDT) were used to obtain multi-unit activity (MUA) and LFP recordings. For MUA recordings, the signals were filtered with a passband of 700 to 7000 Hz, and a threshold was set to retain the spike times of small clusters of units. For LFP recordings, the signals were filtered with a passband of 0.7 to 250 Hz and digitized at 1017.1 Hz.

#### Photo-stimulation

Optogenetic stimulation was done with a 473 nm (blue) laser or with a 470 nm (blue) LED (Omicron Laserage). A 594 nm (yellow) laser was used as control. Laser light was delivered to cortex through a 100 μm or a 200 μm diameter multimode fiber (Thorlabs), LED light through a 2 mm diameter polymer optical fiber (Omicron Laserage). Fiber endings were placed just above the cortical surface, immediately next to the recording sites with a slight angle relative to the electrodes. Laser waveform generation used custom circuits in TDT, and timing control used Psychtoolbox-3, a toolbox in MATLAB (MathWorks) (Brainard, 1997).

For white noise stimulation, the laser was driven by normally distributed white noise, with light intensities updated at a frequency of 1017.1 Hz. For each recording session, the mean of the normal distribution was chosen to fall into the lower half of the dynamic range of the laser-response curve of the recorded MUA. This resulted in mean values in the range of 3-12 mW/mm^2^ (13 MUA recording sites in the 3 cats showing expression of ChR2 in area 17). The standard deviation (SD) of the normal distribution was scaled to be 1/2 the mean. The resulting distributions were truncated at 3.5 SDs. The resulting range of laser intensities always excluded both zero and maximal available laser intensities and thereby avoided clipping.

#### Histology

After conclusion of recordings, approximately five days after the start of the terminal experiment and still under narcosis, the animal was euthanized with pentobarbital sodium and transcardially perfused with phosphate buffered saline (PBS) followed by 4% paraformaldehyde. The brain was removed, post-fixed in 4% paraformaldehyde and subsequently soaked in 10%, 20% and 30% sucrose-PBS solution, respectively, until the tissue sank. The cortex was sectioned in 50 μm thick slices, which were mounted on glass slides in antifade medium, protected with coverslips, and subsequently imaged with a confocal laser scanning microscope (CLSM, Nikon C2 90i, Nikon Instruments) for eYFP-labeled neurons.

#### Immunohistochemistry

In two cats, one with injections in area 17 and one with injections in area 21a, slices were processed as described above and additionally stained for parvalbumin (PV) and gamma-Aminobutyric acid (GABA). To this end, slices were preincubated in 10% normal goat serum (NGS) with 1% bovine serum albumin (BSA) and 0.5% Triton X-100 in phosphate buffer (PB) for 1 h at room temperature to block unspecific binding sites. Floating slices were stained for PV (overnight, rabbit anti-Parvalbumin, NB 120-11427, Novus Biologicals) and GABA (48 hours, rabbit anti-GABA, ABN131, Merck Millipore) in 3% NGS containing 1% BSA and 0.5% Triton X-100. After washing two times 15 min in PB, the slices were incubated with the secondary antibody (goat anti-rabbit Alexa Fluor 647, A-21244, Thermo Fisher Scientific) in 3% NGS containing 1% BSA and 0.5% Triton X-100 for 1 h at room temperature. Finally, slices were again washed in PB, protected with coverslips and imaged with a Zeiss CLSM, using a 25X water immersion objective.

### QUANTIFICATION AND STATISTICAL ANALYSIS

Information about the relevant statistical test can be found in the corresponding results section with additional information concerning data preprocessing and selection in the following Data analysis section. Information about sample variables and size is indicated in the results section, and information pertaining to figure panels can be found in the corresponding figure legend. In general, we applied non-parametric statistical tests, thereby avoiding assumptions about the distributions of our empirical data.

#### Data analysis

All data analysis was performed using custom code and the Fieldtrip toolbox (Oostenveld et al., 2011), both written in MATLAB (MathWorks).

#### Spike densities, MUA-laser cross-correlation, LFP power spectra, and MUA-LFP PPCs

MUA rate was smoothed with a Gaussian (for constant light stimulation: SD = 12.5 ms; for stimulation with pulse trains and sinusoids: SD = 1.25 ms; in each case truncated at ± 2 SD) to obtain the spike density.

To quantify the locking of neuronal responses to optogenetic stimulation, we calculated the Pearson correlation coefficient between MUA spike density and laser intensity as a function of time shift between them.

LFP power spectra were calculated for data epochs that were adjusted for each frequency to have a length of 4 cycles and moved over the data in a sliding-window fashion in 1 ms steps. Each epoch was multiplied with a Hann taper, Fourier transformed, squared and divided by the window length to obtain power density per frequency. For the different stimulation frequencies f, LFP power is shown as ratio of power during stimulation versus pre-stimulation baseline (−0.5 s to −0.2 s relative to stimulation onset).

MUA-LFP locking was quantified by calculating the MUA-LFP PPC (pairwise phase consistency), a metric that is not biased by trial number, spike count or spike rate(Vinck et al., 2010). Spike and LFP recordings were always taken from different electrodes. For each spike, the surrounding LFP was Hann tapered and Fourier transformed. Per spike and frequency, this gave the MUA-LFP phase, which should be similar across spikes, if they are locked to the LFP. This phase similarity is quantified by the PPC as the average phase difference across all possible pairs of spikes. For a given MUA channel, MUA-LFP PPC was calculated relative to all LFPs from different electrodes and then averaged.

#### Estimation of response latency with sinusoidal stimulation

Sinusoidal stimulation of different frequencies enabled estimation of neuronal response latencies. This is highly relevant when optogenetic stimulation is used to produce temporal activation patterns at high frequencies. In addition, it validates that the responses we observe are a result of optogenetic stimulation: Neuronal response latencies to optogenetic stimulation are typically on the order of 3–8 ms; By contrast, shorter latency responses are likely to reflect photo-electric artifacts (Cardin et al., 2010). To investigate response latencies, we averaged MUA responses aligned to the peaks of the sinusoids (Figures S5B–S5F). During sinusoidal stimulation, the light was modulated between the respective maximal intensity and nearly zero intensity. Thus, the light crossed the threshold for effective neuronal stimulation at an unknown intensity, and it is not possible to calculate response latencies in the same way as has been done for pulse trains. Therefore, we used a technique of latency estimation that has been developed in the study of synchronized oscillations, and that is based on the slope of the spectrum of the relative phase between two signals (Schoffelen et al., 2005), in our case the light intensity and the MUA. Figure 3C shows this relative-phase spectrum and reveals a strictly linear relationship between relative phase and frequency. A linear frequency-phase relation is a signature of a fixed time lag, because a given time lag translates into increasing phase lags for increasing frequencies (Schoffelen et al., 2005). The slope of this linear relationship allowed us to infer a latency of 5.5 ms, in good agreement with previous reports of neuronal latencies.

#### Estimation of Granger causality (GC) between light time course and MUA spike trains

The GC spectrum was first estimated separately for each recording site and subsequently averaged over sites. For each trial, we estimated the Fourier transforms of the input (laser) and the output (MUA). Specifically, each trial was segmented into non-overlapping epochs of 500 ms length. Per epoch, the time series of the input and the output were multiplied with a Hann taper, they were zero-padded to a length of 1000 ms, and their Fourier transforms (FTs) were obtained. The FTs were used to calculate the power-spectral densities (PSDs) of the input and of the output, and the cross-spectral density (CSD) between input and output. CSDs and PSDs were averaged over trials and used for the estimation of GC by means of non-parametric spectral matrix factorization (Dhamala et al., 2008). For the example GC spectrum (Figure 4C), the error region was determined by a bootstrap procedure, with 100 iterations, each time randomly choosing 30% of the trials. The shown error boundary is the region containing 95% of the bootstrapped estimates. For the average GC spectrum (Figure 4E), the error region indicates the standard error of the mean across the recording sites.

#### Statistical testing

All inferences were based on the combined data of all animals, for which a given experiment was performed. The resulting inferences are limited to the studied sample of animals, as in most neurophysiological in-vivo studies.

High-resolution spectra of LFP power changes and MUA-LFP PPC were compared between stimulation with blue light and control stimulation with yellow light (Figures 1I and 1J). We calculated paired t tests between spectra obtained with blue and yellow light, across recording sites. Statistical inference was not based directly on the t tests (and therefore corresponding assumptions will not limit our inference), but the resulting t-values were merely used as a well-normalized difference metric for the subsequent cluster-based non-parametric permutation test. For each of 10,000 permutations, we did the following: 1) We made a random decision per recording site to either exchange the spectrum obtained with blue light and the spectrum obtained with yellow light or not; 2) We performed the t test; 3) Clusters of adjacent frequencies with significant t-values (p < 0.05) were detected, and t-values were summed over all frequencies in the cluster to form the cluster-level test statistic. 4) The maximum and the minimum cluster-level statistic were placed into maximum and minimum randomization distributions, respectively. For the observed data, clusters were derived as for the randomized data. Observed clusters were considered significant if they fell below the 2.5^th^ percentile of the minimum randomization distribution or above the 97.5^th^ percentile of the maximum randomization distribution (Maris and Oostenveld, 2007). This corresponds to a two-sided test with correction for the multiple comparisons performed across frequencies (Nichols and Holmes, 2002).

#### PING model

The neurons in the PING model are Hodgkin-Huxley-like point neurons. The excitatory population consists of a simplified version of model pyramidal neurons introduced by (Traub et al., 1991), the reduced Traub-Miles (RTM). The inhibitory population consists of model basket cells introduced by (Wang and Buzsáki, 1996). The parameters for the model are presented in the tables below, and we refer to the original publication of the model for more details (Börgers, 2017).

PING Neuron parameters:

**Table:** 

	C (μF/cm^2^)	v_Na_ (mV)	v_K_ (mV)	v_L_ (mV)	g_Na_ (mS/cm2)	g_K_ (mS/cm2)	g_L_ (mS/cm2)
E (RTM)	1	50	−100	−67	100	80	0.1
I (WB)	1	55	−90	−65	35	9	0.1

PING Network parameters:

**Table:** 

N_E_	200
N_I_	50
I_E_	1.5 μA/cm^2^
σ_E_	0.05 μA/cm^2^
I_I_	0 μA/cm^2^
g_EE_	0 mS/cm^2^
g_EI_	0.25 mS/cm^2^
g_II_	0.25 mS/cm^2^
p_EI_	0.5
p_IE_	0.5
p_II_	0.5
τ_r,E_	0.5 ms
τ_peak,E_	0.5 ms
τ_d,E_	3 ms
V_rev,E_	0 mV
τ_r,I_	0.5 ms
τ_peak,I_	0.5 ms
τ_d,I_	9 ms
V_rev,I_	−75 mV

#### PING+M model

In order to reproduce the experimentally observed hysteresis effects, we implemented spike frequency adaption in the model pyramidal neurons. The PING+M model is taken from the Adaptation-based, Deterministic Weak PING model from Börgers (Chapter 32 of (Börgers, 2017)). In this model, the previous PING model is modified by the addition of a model M-Current to the pyramidal neurons. Otherwise, the network is identical to the PING model described above.

PING+M Neuron parameters:

**Table:** 

g_M_	0.4

#### LIF model

In order to investigate the generality of the model results, we next implemented a simple network of leaky-integrate-and-fire neurons. This network was composed of 80% excitatory neurons and 20% inhibitory neurons, coupled via instantaneous synapses. Excitatory neurons were not mutually connected, while the remaining connectivity was all-to-all, with synapse magnitude randomly distributed uniformly between 0 and the respective post-synaptic-potential (PSP) value. Each neuron accumulates postsynaptic potentials until the threshold for spiking is reached. Upon spiking, each neuron transmits to its synaptic partners a post synaptic event and its potential is reset. The membrane voltage of the model LIF neurons is given by: dV/dt=−V/C+I/C, with the membrane timescale tau = R*C, where R is the input resistance of the neuron, C is the membrane capacitance, and I includes both basal and synaptic currents. We drove the network with symmetric single slow sine waves or with white noise. The dynamics of the network were evaluated numerically at a resolution of tau using the Euler method.

V=V+dt∗(−(V−E)+I∗R)/tau

LIF Network parameters:

**Table:** 

N_E_	200
N_I_	50
I_E_	1.5 nA
σ_E_	0.05 μA
I_I_	0 μA
PSP_EE_	1.1 mV
PSP_IE_	0.6 mV
PSP_EI_	−0.8 mV
PSP_II_	−0.8 mV
Dt	0.5 ms
C	0.6 nF
R	40 Mohms
V_spike_	30 mV
V_thresh_	−40 mV
V_reset_	−75 mV
V_leak_	−60 mV

## Supplementary Material

1

## Figures and Tables

**Table:** KEY RESOURCES TABLE

REAGENT or RESOURCE	SOURCE	IDENTIFIER
Bacterial and virus strains		
AAV5 ChR2	UNC Vector core	AAV5-CamKIIα-ChR2-eYFP
AAV1 ChR2	UNC Vector core	AAV1-CamKIIα-hChR2(H134R)-eYFP
AAV9 ChR2	Penn Vector core	AAV9-CamKIIα-hChR2(H134R)-eYFP
Experimental models: Organisms/strains		
*Felis catus*	In house	NA
Software and algorithms		
OpenX	Tucker Davis	NA
MATLAB	Mathworks	https://www.mathworks.com/products/matlab.html
FieldTrip	Oostenveld et al., 2011	https://www.fieldtriptoolbox.org/

## Highlights

- Optogenetics is used to emulate synaptic input under precise temporal control
- The cortical microcircuit shows resonance in the gamma-frequency band
- Slow input variation modulates gamma frequency and reveals hysteresis
- White-noise input reveals preferential transmission of resonance-coherent input
