# Supplementary material for: Cortical gamma-band resonance preferentially transmits coherent input
Source: Cell Rep. Author manuscript; Available in PMC 2021 Jun 14. (PMC8200519; doi:10.1016/j.celrep.2021.109083)
Supplement: 1 [file NIHMS1700575-supplement-1.pdf]

Cell Reports, Volume *35*

Supplemental information

Cortical gamma-band resonance

preferentially transmits coherent input

Christopher Murphy Lewis, Jianguang Ni, Thomas Wunderle, Patrick Jendritza, Andreea Lazar, Ilka Diester, and Pascal Fries

Supplemental Figure S1:

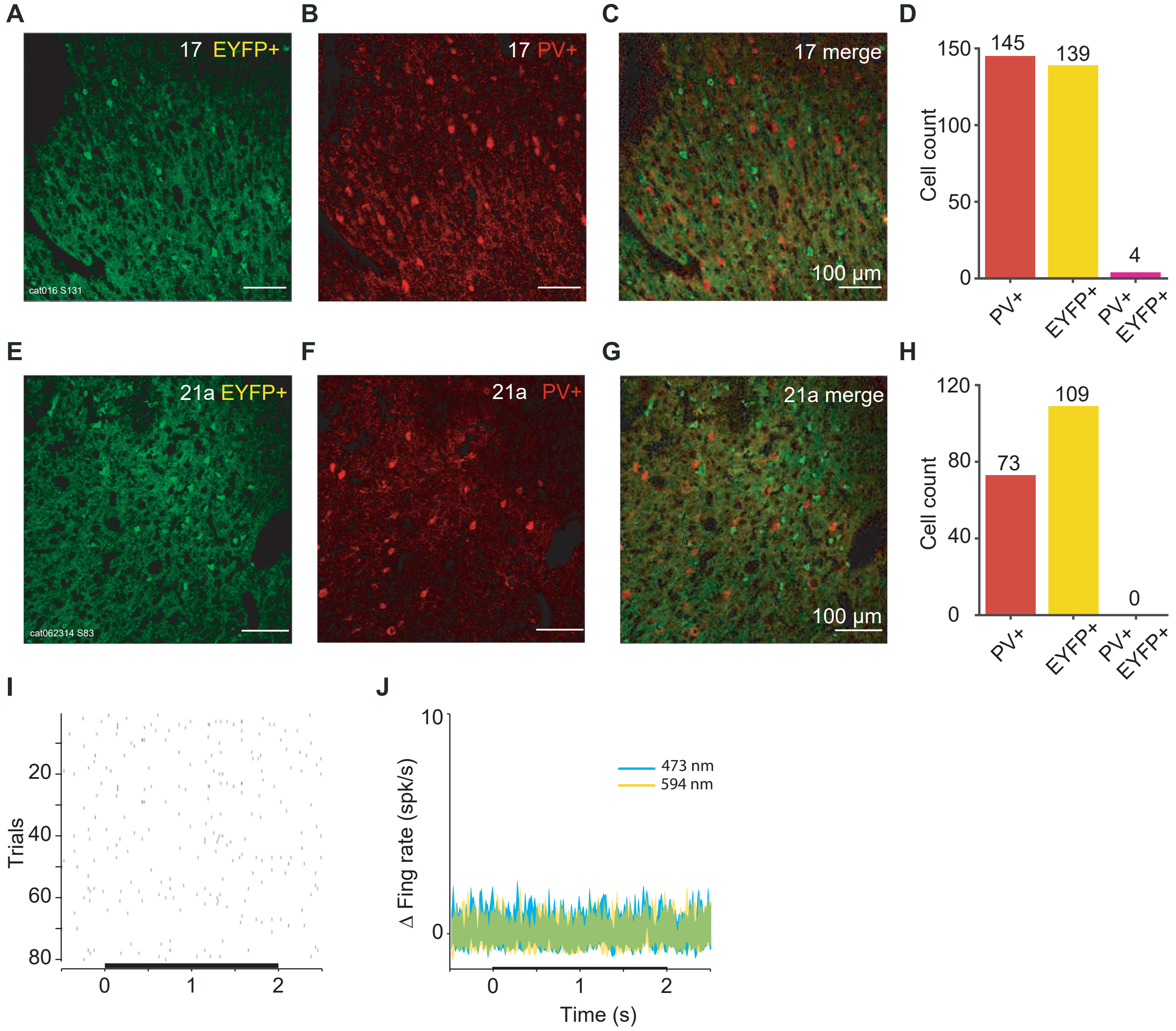

Supplemental Figure S1, related to Figure 1. Viral transfection was largely selective for excitatory neurons. (A-C) Confocal images of immunohistochemistry performed on slices from area 17 after viral transfection. (A) Endogenous fluorescence of ChR2-eYFP. (B) Fluorescence of secondary antibody after staining for PV+. (C) Merged images, testing for neuronal co-labeling with ChR2-eYFP and PV+ antibody. (D) Counts of PV+ labeled neurons, EYFP+ labeled neurons, and co-labeled neurons in area 17. (E-H) Same as A-D, but for area 21a. No co-labeled neurons can be found. (I) Example MUA response to 2 s of blue laser stimulation to a site in area 21a not expressing ChR2. (J) Group results showing firing rate changes from baseline for 2 s of constant blue and yellow laser stimulation for 8 sites (5 area 21a, 3 area 17) in 4 cats.

Supplemental Figure S2:

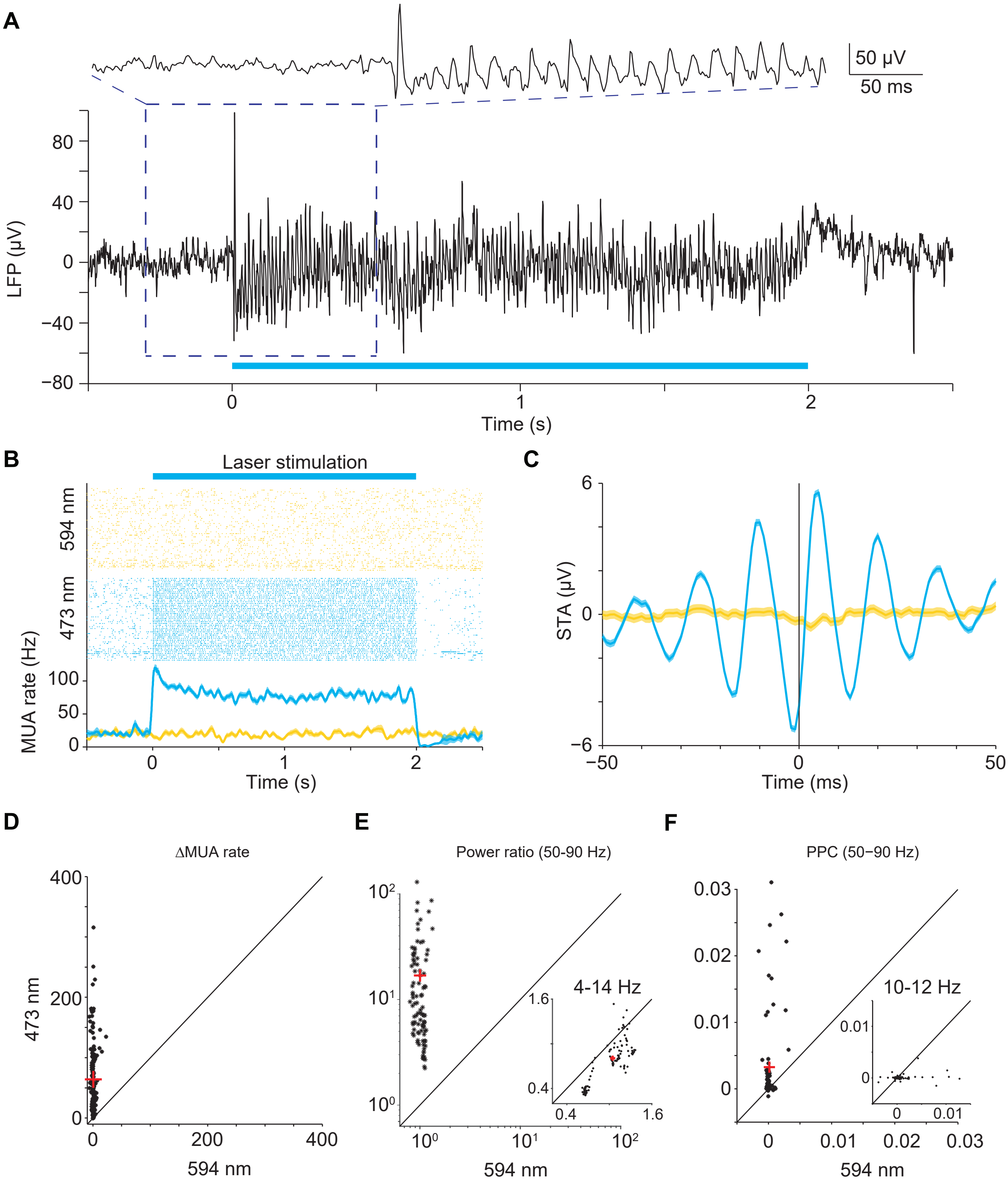

Supplemental Figure S2, related to Figure 1. Group results for MUA rate, LFP power and MUA-LFP PPC induced by constant light. (A) Example recording site in area 21a shows strong gamma-band synchronization in the local field potential induced by constant-illumination. (B) Robust MUA response to constant illumination at the same site. (C) Spike-triggered LFP for example data shown in A and B. (B-C) Blue (yellow) lines show data obtained with 473 nm (594 nm) light stimulation. Shaded areas indicate ±1 SEM across recording sites, which is shown for illustration only. For the main clusters from Figure 1 panels (H-J), panels (D-F) illustrate the underlying distributions as scatter plots. (D) Each dot shows the MUA rate (0.3-2 s after light onset) of one recording site for blue light on the y-axis versus yellow light at the x-axis. The red cross corresponds to the respective median values. (E) Same as (D), but for the LFP power ratio. The main plot is for the gamma band (50-90 Hz); the inset plot for the low-frequency cluster from (Figure 1I) (4-14 Hz). (F) Same as (D), but for MUA-LFP PPC during light stimulation. Each dot corresponds to one MUA recording site. The main plot is for the gamma band (50-90 Hz); the inset plot is for the low-frequency cluster from (Figure 1J) (10-12 Hz).

Supplemental Figure S3:

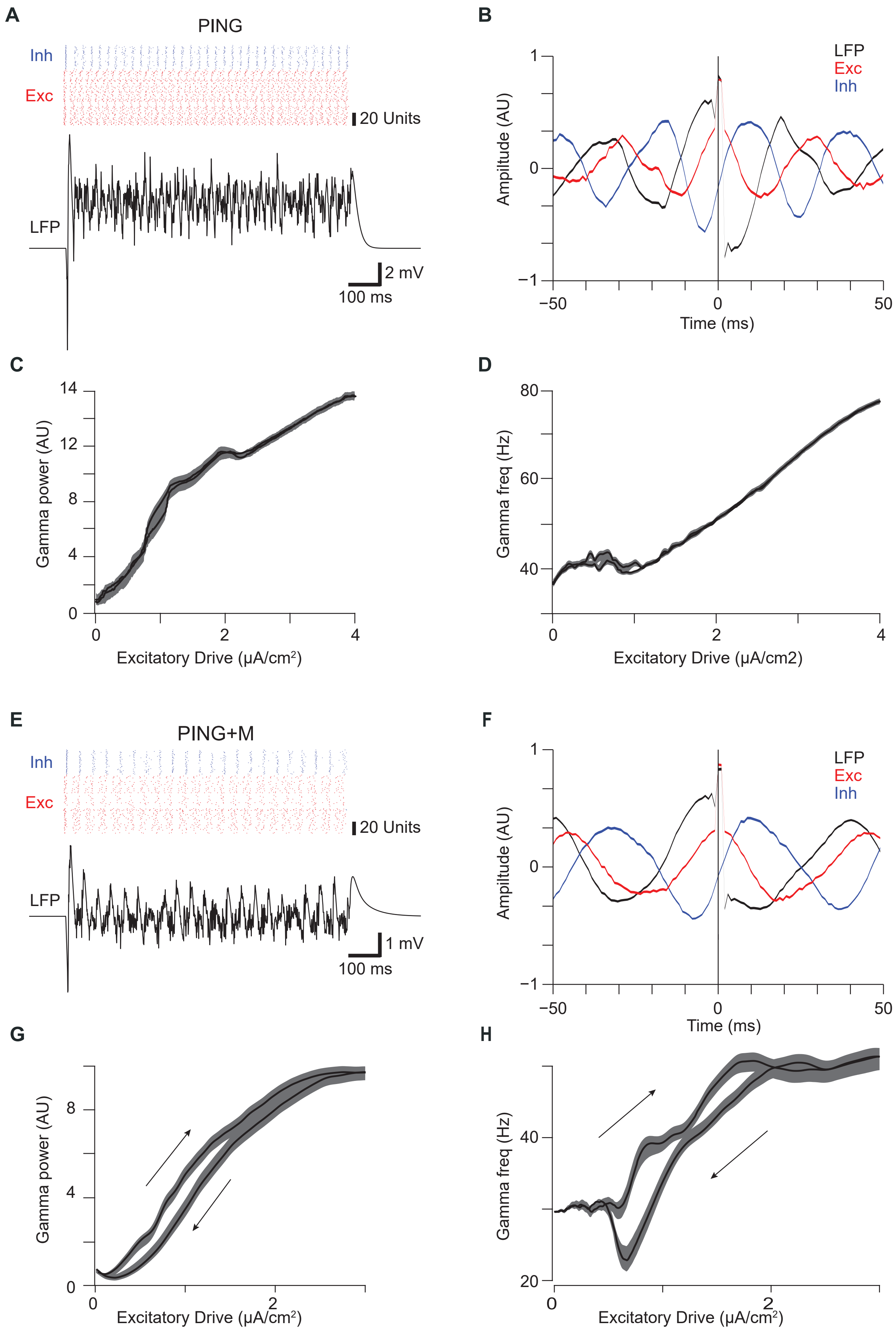

Supplemental Figure S3, related to Figure 2. Synchronization in the PING and PING+M models. (A-B) Response of the PING model to constant stimulation. (A) Raster plot of inhibitory (Inh) and excitatory (Exc) neuron spiking, and average membrane potential (LFP) reveal robust gamma-band synchronization in the PING model in response to constant excitation. (B) Spike-triggered averaging, based on spikes of excitatory units, in the PING model reflects characteristic gamma cycle with excitation leading inhibition. (C) Gamma power and (D) frequency increase with increasing excitatory drive in the PING network, but do not demonstrate hysteresis. (E-F) Response of the PING+M model to constant stimulation. (E) Raster plot of inhibitory (Inh) and excitatory (Exc) neuron spiking, and average membrane potential (LFP) reveal robust gamma-band synchronization in the PING+M model, but at a lower frequency as compared to the PING model, when constant excitation is matched. (F) As in (B), but for the PING+M model. (G) In the PING+M model, gamma power and (H) frequency increase with increasing excitatory drive. Arrows indicate hysteresis in response to increasing (upper arrow) versus decreasing (lower arrow) laser power, in qualitative agreement with recordings.

Supplemental Figure S4:

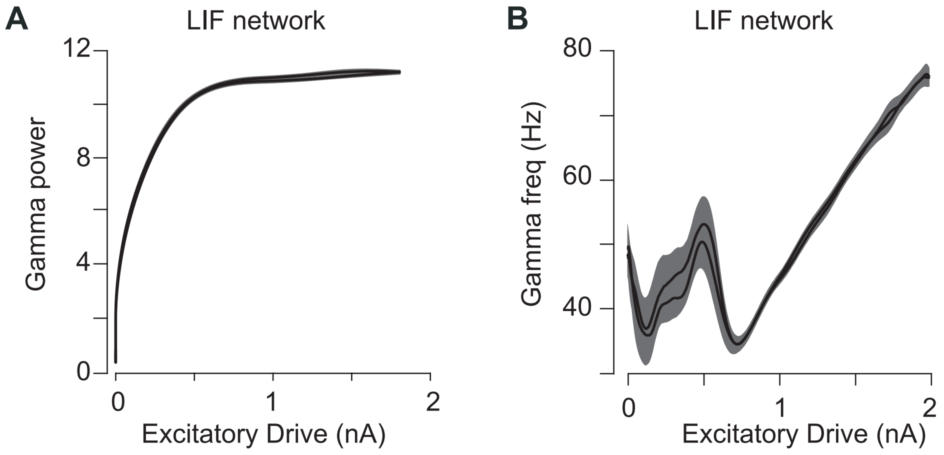

Supplemental Figure S4, related to Figure 2. Synchronization of LIF network with increased excitatory drive. (A-B) LIF network exhibits increased gamma power (A) and frequency (B) with increased excitatory drive, but does not display hysteresis.

Supplemental Figure S5:

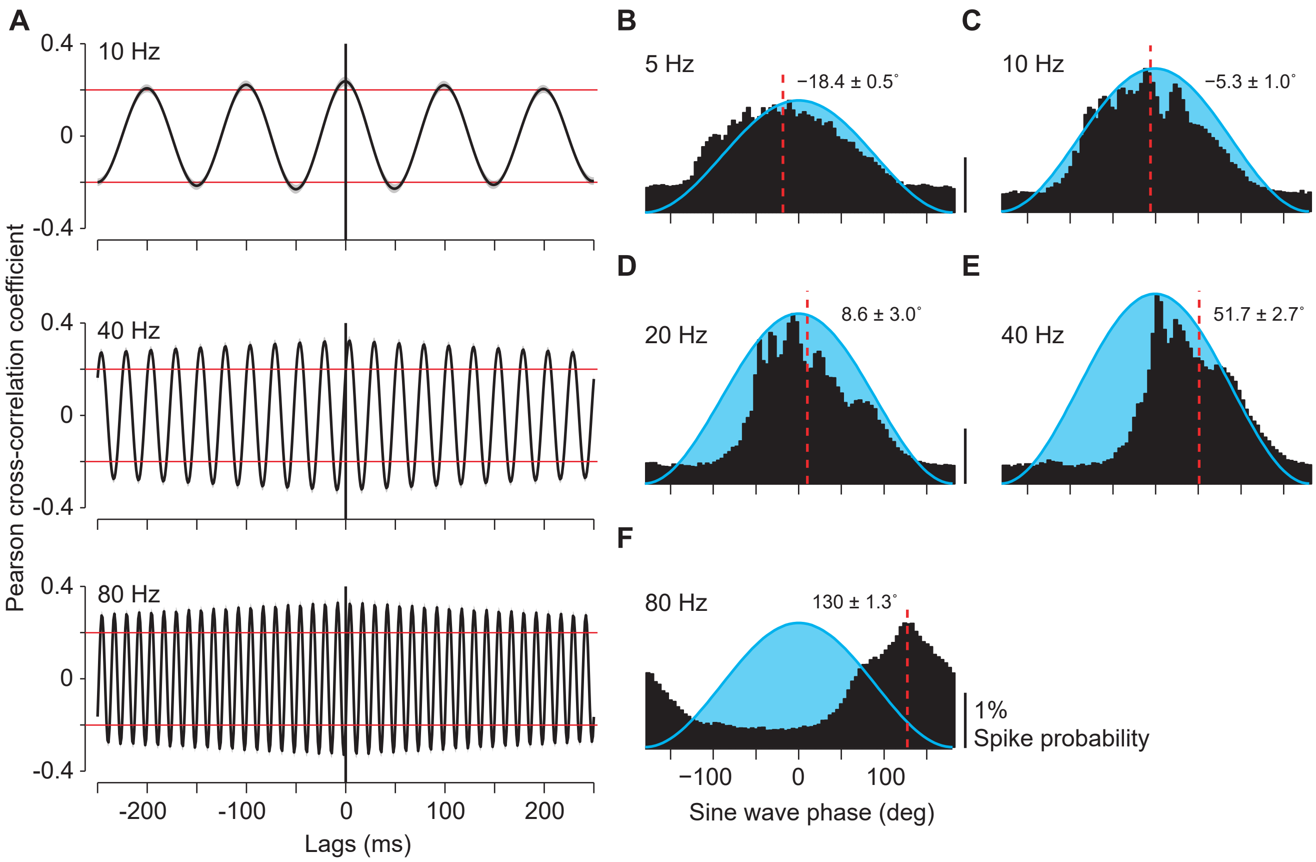

Supplemental Figure S5, related to Figure 3. MUA responses to sinusoidal stimulation. (A) The Pearson cross-correlation coefficients between sinusoidal optogenetic drive and MUA for stimulation at 10 Hz, 40 Hz and 80 Hz demonstrate increased correlation in the gamma-band. Red horizontal lines are shown at ±0.2 in all panels to easy comparison. (B-F) MUA spike probability, averaged over recording sites, as a function of the phase of the optogenetic sine wave stimulation. The optogenetic sine wave is indicated by the blue-shaded region. Each panel shows the data obtained with the frequency indicated on top of the panel. MUA responses were fitted with Gaussians, and the resulting peak latencies are indicated by dashed red lines. Peak latencies and their SEM (estimated through a jackknife procedure) are indicated as text insets. Latencies are expressed relative to the time of peak light intensity.

Supplemental Figure S6:

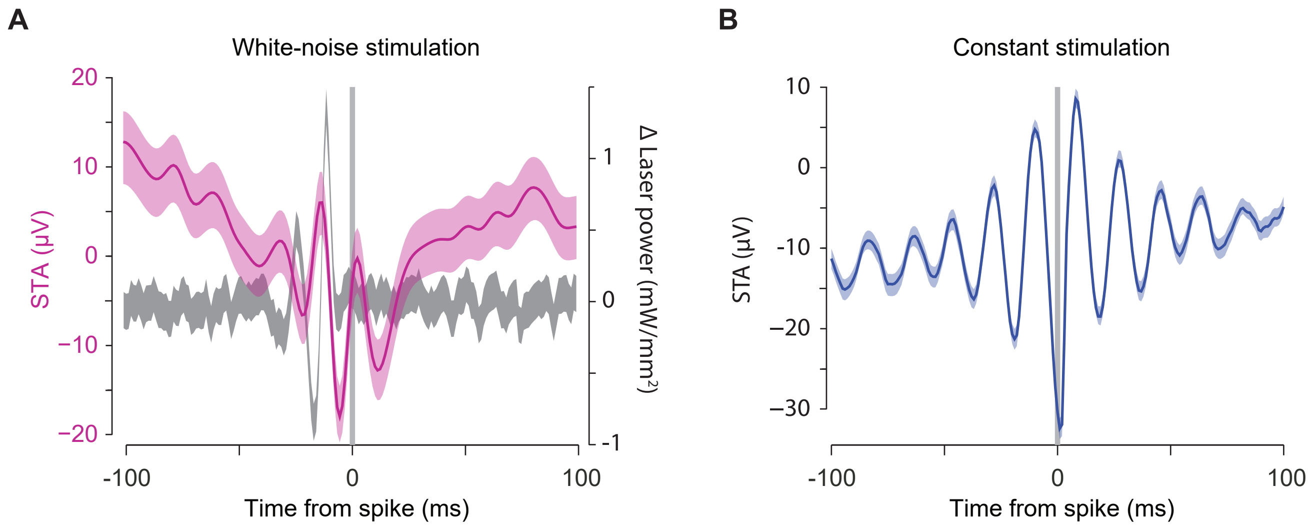

Supplemental Figure S6, related to Figure 4. Time scale of gamma-band synchronization during white-noise and constant stimulation. (A) Spike-triggered white noise (grey) and spike-triggered LFP (red), during white-noise stimulation. (B) Spike-triggered LFP for constant optogenetic stimulation of the same average laser power as in (A).

Supplemental Figure S7:

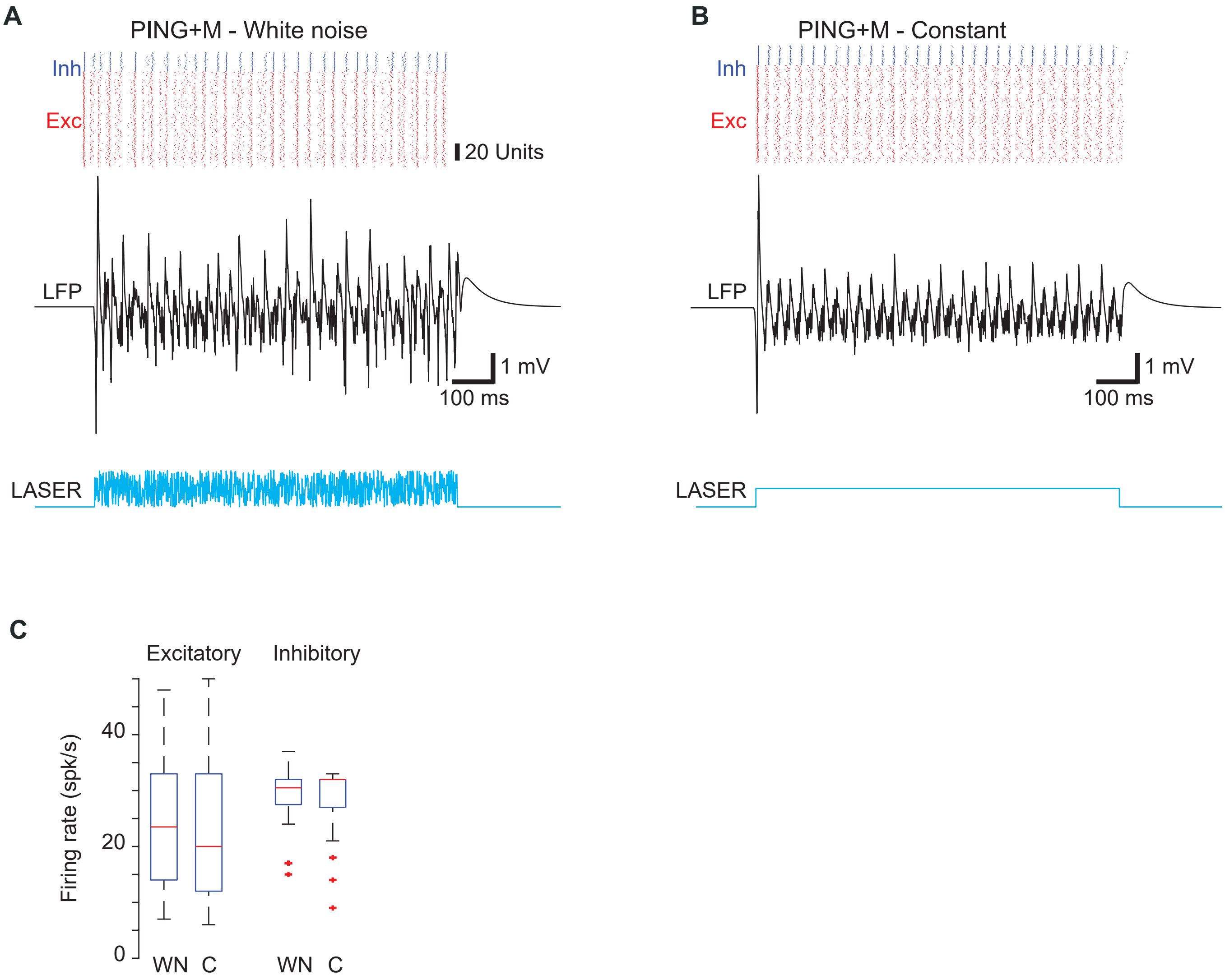

Supplemental Figure S7, related to Figure 5. Synchronization in the PING+M model. (A) Response of the PING+M model to white noise stimulation. Raster plot of inhibitory (Inh) and excitatory (Exc) neuron spiking, and average membrane potential (LFP) reveal robust gamma-band synchronization in the PING model in response to constant excitation. (B) Same as in A, but for constant stimulation. (C) Comparison of the firing rates of excitatory and inhibitory units in the PING+M model when driven with white noise or constant stimulation.

Supplemental Figure S8:

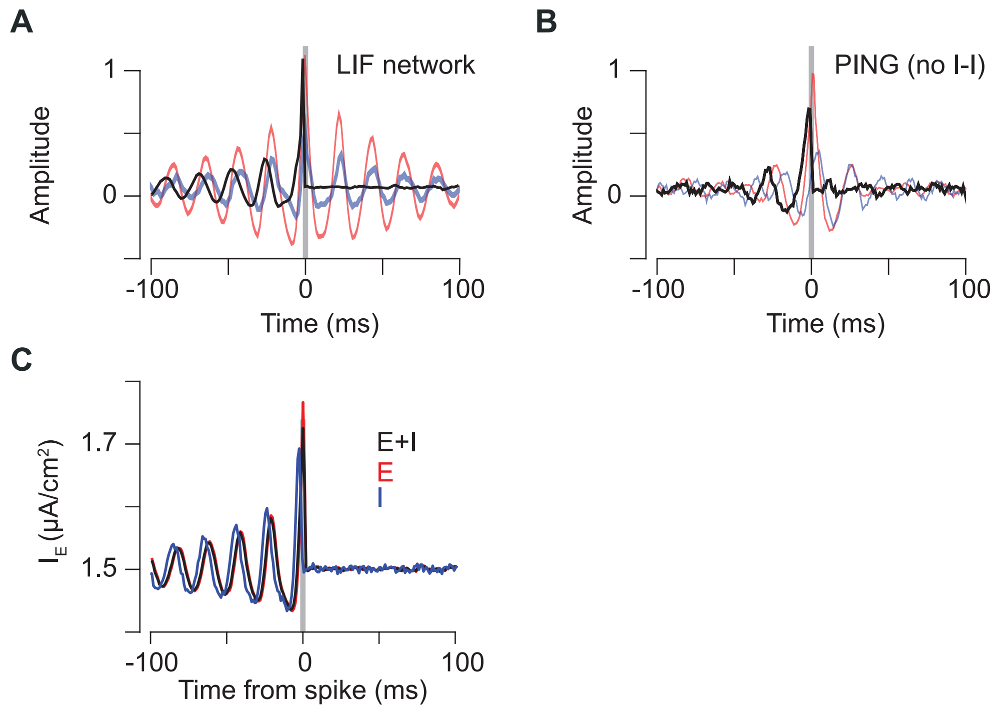

Supplemental Figure S8, related to Figure 5. Selective transmission in LIF network and in PING network without I-I connectivity. (A) LIF network exhibits selective transmission of coherent input. (B) PING network without I-to-I coupling exhibits selective transmission of coherent input, with reduced effect strength and lower frequency. (C) Spike-triggered-average of white-noise for different components of the PING network: all units (black), excitatory units (red), and inhibitory units (blue).
